# The Effect of Salinized Nano ZrO_2_ Particles on the Microstructure, Hardness, and Wear Behavior of Acrylic Denture Tooth Nanocomposite

**DOI:** 10.3390/polym14020302

**Published:** 2022-01-12

**Authors:** Kawkb M. El-Tamimi, Dalia A. Bayoumi, Mohamed M. Z. Ahmed, Ibrahim Albaijan, Mohammed E. El-Sayed

**Affiliations:** 1 Department of Removable Prosthodontics, Faculty of Dentistry, Suez Canal University, Ismailia 41522, Egypt; kowkb_eltamimi@dent.suez.edu.eg (K.M.E.-T.); mohamed_ezzat@dent.suez.edu.eg (M.E.E.-S.); 2 Department of Dental Biomaterials, Faculty of Dentistry, Suez Canal University, Ismailia 41522, Egypt; dalia_bayoumi@dent.suez.edu.eg; 3 Mechanical Engineering Department, College of Engineering at Al Kharj, Prince Sattam Bin Abdulaziz University, Al Kharj 16273, Saudi Arabia; i.albaijan@psau.edu.sa; 4 Department of Metallurgical and Materials Engineering, Faculty of Petroleum and Mining Engineering, Suez University, Suez 43512, Egypt

**Keywords:** nanocomposite, acrylic denture teeth, nano ZrO_2_ particles, wear behavior, microstructure, microhardness, polymerization, polymethyl methacrylate resin

## Abstract

The wear of acrylic denture teeth is a serious problem that can change the vertical dimensions of dentures. This study evaluates the effect of adding salinized nano ZrO_2_ particles on the microstructure, hardness, and wear resistance of acrylic denture teeth. Heat polymerizing polymethyl methacrylate resin was mixed with salinized ZrO_2_ at concentrations of 5 wt.% and 10 wt.%. Acrylic resin specimens without filler addition were used as a control group. SEM/EDS analyses were performed and the Vickers’ hardness was evaluated. Two-body wear testing was performed using a chewing simulator with a human enamel antagonist. After subjecting the samples to 37,500 cycles, both height loss and weight loss were used to evaluate the wear behavior. The microstructural investigation of the reinforced-denture teeth indicates sound nanocomposite preparation using the applied regime without porosity or macro defects. The addition of zirconium oxide nanofillers to PMMA at both 5% and 10% increased the microhardness, with values of up to 49.7 HV. The wear mechanism in the acrylic base material without nanoparticle addition was found to be fatigue wear; a high density of microcracks were found. The addition of 5 wt.% ZrO_2_ improved the wear resistance. Increasing the nanoparticles to 10 wt.% ZrO_2_ further improved the wear resistance, with no microcracks found.

## 1. Introduction

Denture teeth are currently made of either methacrylate-based resins (acrylic resin) or porcelain, but acrylic teeth have nearly eliminated porcelain teeth from the market [1] due to a number of advantages, including their chemical bonding to the denture base [2,3], lower susceptibility to fracture [4,5] and decreased clicking [6,7]. Acrylic resin tooth wear is a serious complication during denture service and can change the vertical dimension of dentures. This process harms the denture and exerts an impact on facial aesthetics and the function of the masticatory muscles, resulting in less efficient mastication. This can result in temporomandibular disorders, digestive disturbances and decreased patient comfort [6,7,8,9]. Efforts were made to enhance the wear resistance of the acrylic resin denture teeth, such as the formation of cross-linked polymer teeth (interpenetrated polymer network) [8,9]. Another possible solution is to add nanofillers to enhance mechanical properties. Despite this, these materials are softer than composite resin or porcelain teeth [8,9,10]. Nano-fillers, such as metal oxides, carbon, and glass fibers have also been used to improve the mechanical behavior of the acrylic resin denture base materials [11,12,13,14,15,16]. Recently, zirconium oxide nanoparticles (ZrO_2_ NPs) have been recognized for their high biocompatibility. However, because they are white in color, they are thought to be less likely to change aesthetics than other metal oxide nanoparticles [17]. ZrO_2_ NPs are not only biocompatible but are also resistant to wear and corrosion. In addition to these characteristics, ZrO_2_ NPs offer high toughness and mechanical strength [18,19,20]. ZrO_2_ NPs are frequently used to mechanically reinforce polymers [12]. Saline coupling agents are applied to the surface of ZrO_2_ NPs to decrease the risk of aggregation and enhance compatibility with the polymer matrix [21]. The stresses caused by the dispersion of ZrO_2_ NPs are transferred from the weak Polymethylmethacrylate (PMMA) matrix to the strong nanoparticles [20]. Ayad et al. [18] reported a minor increase in the surface hardness and the impact strength of zirconium dioxide acrylic composite when compared to the unreinforced resin (control specimen). Another study reported a reduction in impact strength as well as surface hardness when 10 wt.% ZrO_2_ and 20 wt.% ZrO_2_ were added [22]. Alternatively, a study found that increasing the amount of modified (ZrO_2_) added to nano-ZrO_2_/PMMA composite increased the hardness by up to 10%, and increased the hardness of groups 5% and 10% (water storage groups) compared with the zero and 20% (*p* < 0.01). The highest hardness value was observed in group 5% (TC− 36.8, TC+ 35.2) [23,24]. Furthermore, after incorporating salinized zirconia NPs in acrylic resin, there was a significant increase in hardness and a minor improvement in surface roughness, as well as a decrease in porosity [25,26]. However, investigations into the addition of salinized nano-ZrO_2_ powder to acrylic resin teeth are lacking. The overall aim of this study is to develop nanocomposite denture teeth with high wear resistance. To this end, the effect of incorporating salinized nano-ZrO_2_ particles into acrylic resin on the acrylic denture tooth microstructure, hardness, and wear resistance was investigated.

## 2. Materials and Methods

### 2.1. ZrO_2_ Nanoparticles Surface Treatment 

To enhance the ZrO_2_ nanoparticle’s wettability with the resin matrix through the formation of a reactive group on their surface, the salinization process was carried out in the laboratory of Inorganic Chemistry, Chemistry Department, Faculty of Science, Suez Canal University. The ZrO_2_ nanoparticle powder of 99.9% purity, with 9 ± 2 m^2^/g surface area and an average particle size of 40 ± 3 nm, (Nanogate, Cairo, Egypt) was saline-treated using the saline coupling agent 3-trimethoxysilyl propylmethacrylate (TMSPM) (Sigma-Aldrich, Berlin, Germany) [27]. This procedure was accomplished by mixing 30 g of ZrO_2_ nanoparticles with 0.3 g of TMSPM dissolved in 100 mL of acetone for 1 h with the help of a magnetic stirrer at 300 rpm (Hot plate with a magnetic stirrer, MSH-A 30A, Seoul, Korea) for 60 min. Subsequently, the solvent was removed using a rotary evaporator under vacuum for 30 min at 60 °C and 150 rpm, followed by heating for 2 h at 120 °C. Next, the treated powder was allowed to cool to 28 °C [28,29,30].

### 2.2. PMMA Powder of Artificial Teeth/ZrO_2_ Nanocomposite Preparation

**Figure 1:**
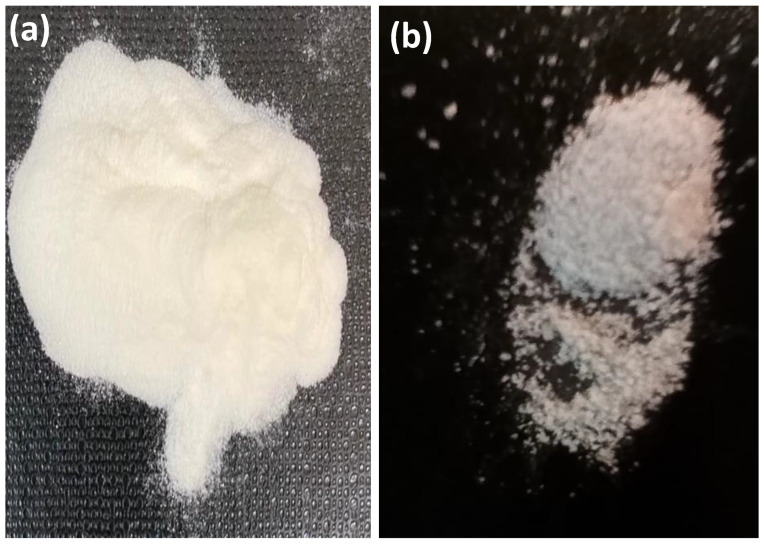
Macro images for the raw material powder used in this work (**a**) acrylic resin powder of artificial teeth and (**b**) ZrO_2_ powder.

The salinized nano-Zirconium oxide particles and PMMA powder of artificial teeth (Pigeon Dental Co Shanghai, Beijing, China) (Figure 1a) were pre-weighed using an electronic balance (Sartorius, Done Biopharmaceutical and Laboratories, Berlin, Germany) with an accuracy of 0.0001 gm so that the Nano-filler (Figure 1b) concentrations were 5% and 10% by weight. Pre-weighed Nano-filler ZrO_2_ powder with two concentrations (5% and 10%) was added individually to the heat polymerized PMMA powder of artificial teeth and thoroughly mixed using an electric mixer to produce a uniform blend and ensure that the homogeneous mix of PMMA/ZrO_2,_ was mixed independently [18,20,29,31,32]. 

**Figure 2:**
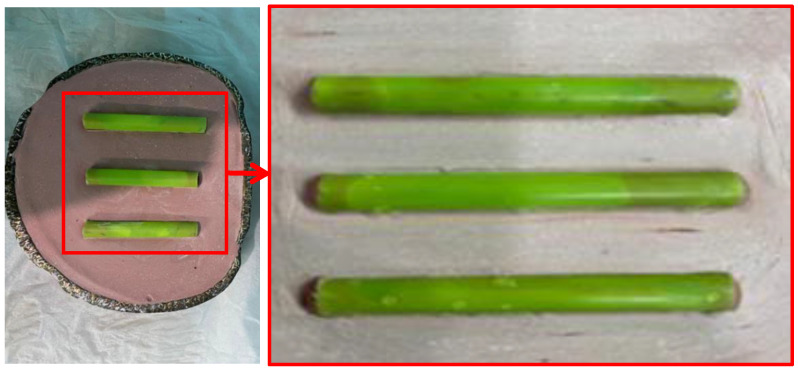
An image for the metal flask with the wax specimens invested and a magnified region of the wax specimen.

Twenty-four cylindrical samples 4 mm in diameter × 8 mm in height, were prepared from acrylic resin of teeth material. The control group (GI) was made from the conventional PMMA without zirconium oxide nano-particles. Two other groups GII, GIII, featured concentrations of 5 and 10 wt.% of zirconium oxide reinforced acrylic resin tooth-forming material. These samples were used to evaluate the wear behavior by using the chewing simulator and also to evaluate the hardness. Wax cylinders, of 8 mm × 100 mm shown in Figure 2, (Cavex, Haarlem, The Netherlands) were used for wax pattern fabrication that invested in a metal flask with dental stone (Zeta Mufle, Nevilicure, Italy).

After setting the stone, the wax was melted by placing the flasks in a wax elimination machine for 10 min. After removing the softened wax and all wax traces, a separating medium (Acrostone(A), Anglo-Egyptian Company. Hegaz, Cairo, Egypt, Batch No.505/04) was applied on the warm stone mold surfaces. When the flasks cooled to room temperature, heat-polymerized resin (pure or reinforced with nano-ZrO_2_) was mixed and packed into the mold spaces at the dough stage. The flask parts were brought together under pressure using a hydraulic bench press for 5 min and then allowed to rest for 30 min. To polymerize the resin, the flasks were placed in a laboratory curing bath for 90 min at 74 °C, and then 30 min at 100 °C. After curing, the flasks were left for slow bench cooling till 28 °C before opening. Acrylic samples were retrieved from the stone, finished, and polished. Initially, the excess resin was trimmed using progressively finer grits of silicon carbide papers (grits 120–500) in wet conditions, followed by polishing using a rag wheel and pumice on a dental lathe and felt disc on a polishing machine, and the samples were cut to the required dimensions (4 × 8 mm). A digital micrometer (Mitutoyo, Digimatic Caliper 25SB, Mitutoyo Corporation, Tokyo, Japan) with a precision of 1/1000 was used to measure specimen dimensions [27]. An electronic balance (Sartorius, Biopharmaceutical and Laboratories, Berlin, Germany) with an accuracy of 0.001 gm was used to measure samples weight before and after the wear test. 

### 2.3. Micro-Hardness Test Procedure 

Micro-hardness of all the acrylic samples was investigated using Digital Display Vickers Micro-hardness Tester (Model HVS-50, Laizhou Huayin Testing Instrument Co., Ltd. Beijing, China) using a Vickers diamond indenter with a 20× objective lens. A load of 0.98 N was applied to the surface of the specimens for a 10 s dwell time. Three indentations, which were equally placed over a circle and not closer than 0.5 mm to the adjacent indentations, were made on the surface of each sample. The diagonal length of the indentations was measured by a built-in scaled microscope and Vickers micro-hardness values were obtained [30]. The micro-hardness mean value was then calculated for each sample. The micro-hardness was obtained using Equation (1).

HV *=* 1.854 *P/d*^2^ (1)

where HV is Vickers hardness in Kgf/mm^2^, *P* is the load in Kgf, and *d* is the length of the diagonals in mm.

### 2.4. Wear Testing with Natural Teeth Surface Antagonist 

**Figure 3:**
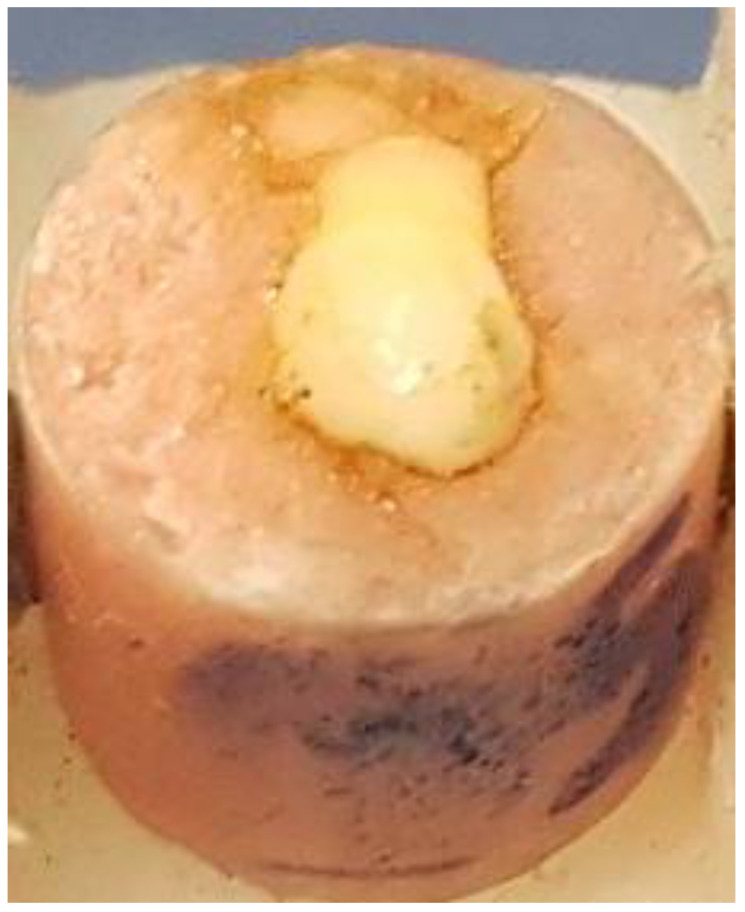
Image of assembly of the natural teeth within the self-cured acrylic resin mold.

Carie-free human premolar teeth without fractures or worn cusps that were recently extracted for orthodontic treatment plans were chosen for this study and stored in 0.1% thymol solution (approved by the accredited ethics committee of the Faculty of Dentistry Suez Canal University, no297/2020). The buccal cusps with buccal surface were cut using no. 943 Miniflex diamond disc (Brasseler, Lemgo, Germany). They were then inserted into a self-cured acrylic resin mold (Acrostone Co., Cairo, Egypt) inside copper specimen holder with diameter15 mm. The insertion of the natural tooth surfaces was guided by a dental surveyor to ensure proper alignment of the tooth surface to the long axis of the specimen holder [33,34]. Figure 3 shows the assembly of the natural teeth within the self-cured acrylic resin mold.

**Figure 4:**
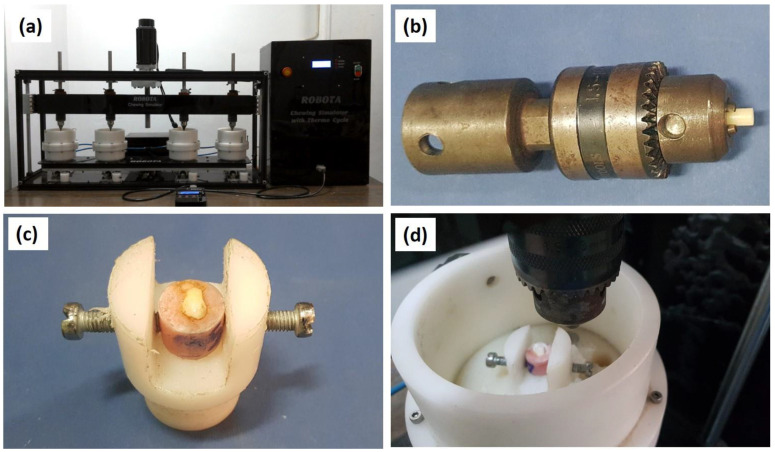
Images of the wear testing stages: (**a**) chewing simulator setup, (**b**) fixing the acrylic teeth sample in the holder, (**c**) plastic holder of the device acting as the antagonist, (**d**) positioning of the upper samples holder in point contact with the teeth surfaces.

As a preparation for the wear test, the buccal surface of each tooth specimen was wet abraded and finished with grit abrasive paper (1000, 2500, and 4000), to a total depth of 0.5 mm. As a result, a flat area of about 2–3 mm was obtained for loading during the wear test [34,35]. Next, the two-body wear test was performed using the newly developed four stations multimodal Dual-axis ROBOTA chewing simulator integrated with thermo-cyclic protocol operated on servo-motor (Model ACH-09075DC-T, AD-TECH Technology Co., LTD., Berlin, Germany). The device allows the simulation of vertical and horizontal movements simultaneously in the thermodynamic condition in a range of 5 °C to 55 °C with a dwell time of 10 s. The ROBOTA chewing simulator features four chambers, each consisting of an upper part as a sample holder and a lower plastic antagonist holder Figure 4a. Samples were mounted into the metal receptacle present in the chewing simulator upper part Figure 4b. All the samples were tested under standard conditions in which the buccal surface of premolar teeth was used in the lower plastic holder of the device acting as antagonist Figure 4c. The acrylic samples were positioned on the upper samples holder in point contact with the teeth surfaces Figure 4d. A weight of 5 kg, which is comparable to 49 N of chewing force, was exerted. The samples were subjected to 37,500 cycles to simulate three months of clinical function. After 37,500 cycles, the samples were removed from the holder, cleaned with running water, and followed by cleaning in an ultrasonic cleaner for 2 min to remove any abraded particles from the surface before measurements.

Both height loss and weight loss were used to evaluate the wear behavior of the pure PMMA- and ZrO_2_-reinforced tooth samples. Height loss was measured using the digital micrometer (Mitutoyo, Digimatic Caliper 25SB, Mitutoyo Corporation, Tokyo, Japan) with a precision of 1/1000. The difference between the readings before and after the wear simulation gave the amount of vertical sample height loss. Furthermore, weight loss was measured by weighing samples in the electronic analytical balance (Sartorius, Biopharmaceutical and Laboratories, Berlin, Germany) with an accuracy of 0.001 gm to show the difference in weight before and after the wear test. As this electronic balance featured a fully automated calibration technology and a micro weighing scale, values of all the mounted discs and antagonist samples were accurately measured. Each mounted sample was cleaned and dried with tissue paper before weighing. All data were calculated, tabulated, and statistically analyzed using SPSS for Windows, version 22.0 (Statistical Package for Social Science, Armonk, NY, USA: IBM Corp) at significant levels 0.05 (*p*-Value ≤ 0.5). A normality test was done to check the normal distribution of the sample, and all groups and subgroups showed normal distributions.

(A) Descriptive data: Descriptive statistics were calculated in the form of Mean ± Standard deviation (SD), median, range (Max-Min).
(B) ANOVA—test: One-way ANOVA (Analysis of variance) was used to compare between the three groups under study. Tukey Post hoc test was performed for the evaluation of statistical significance among the groups. *p*-value ≤ 0.05 is considered to be statistically significant.

### 2.5. Microstuctur Investigation

The microstructure of the acrylic nanocomposites was investigated using optical microscopy (OM) and scanning electron microscopy (SEM) equipped with EDS analysis. For the microstructural analysis, the samples were prepared using grinding with conventional silicon carbide emery papers of different grit sizes and then mechanically polished using alumina suspension of 0.05 µm [36,37]. Furthermore, the samples were investigated with SEM after the wear testing to study the worn surface and determine the wear mechanisms. 

## 3. Results 

### 3.1. Acrylic/ZrO_2_ Nanocomposites Microstructure 

**Figure 5:**
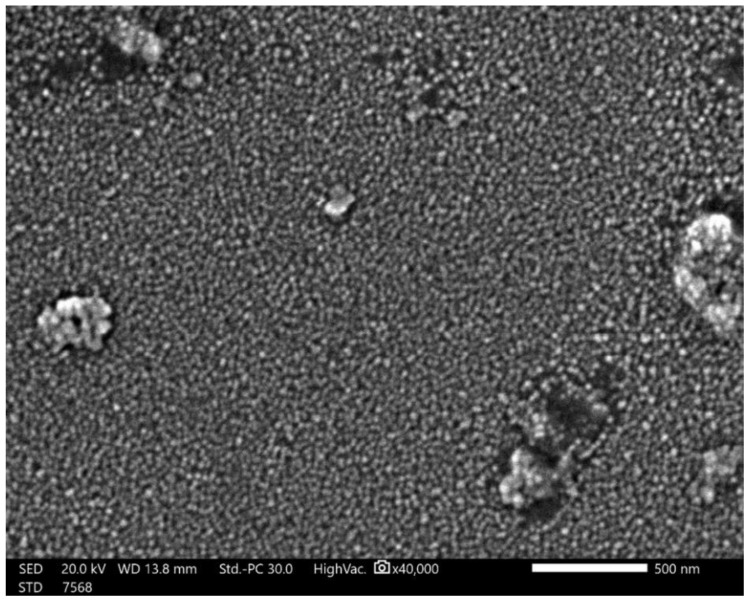
SEM micrograph for the raw material powder of the ZrO_2_ nanoparticles used as a reinforcing material for the acrylic matrix.

**Figure 6:**
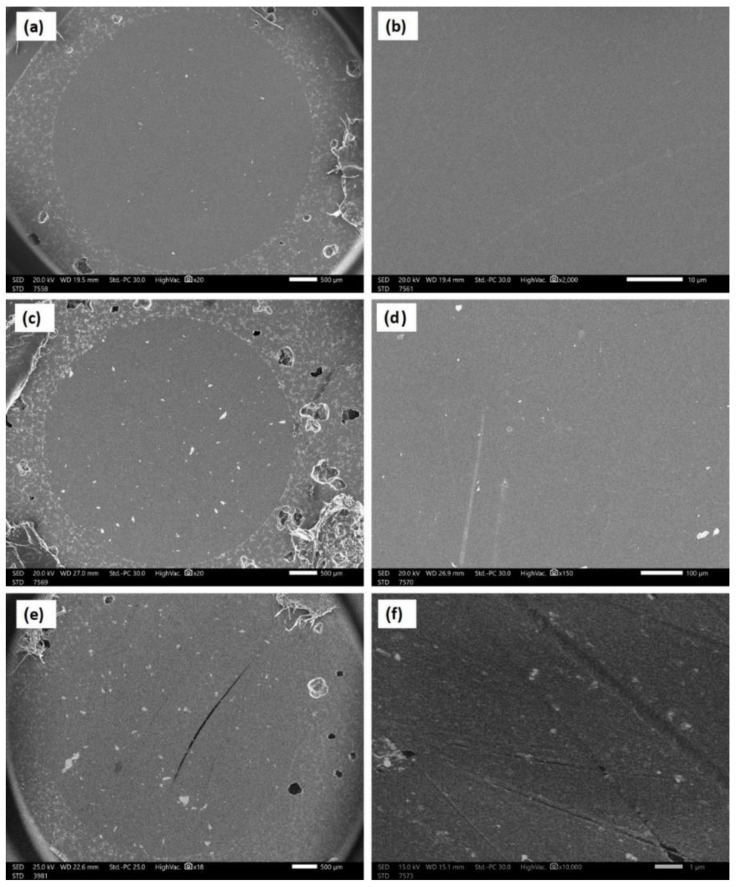
SEM macro and micrographs of the base acrylic material(**a**,**b**), (**c**,**d**) 5 wt.% ZrO_2_ nano-composites (**c**,**d**) and 10 wt.% ZrO_2_ nano-composites (**e**,**f**).

**Figure 7:**
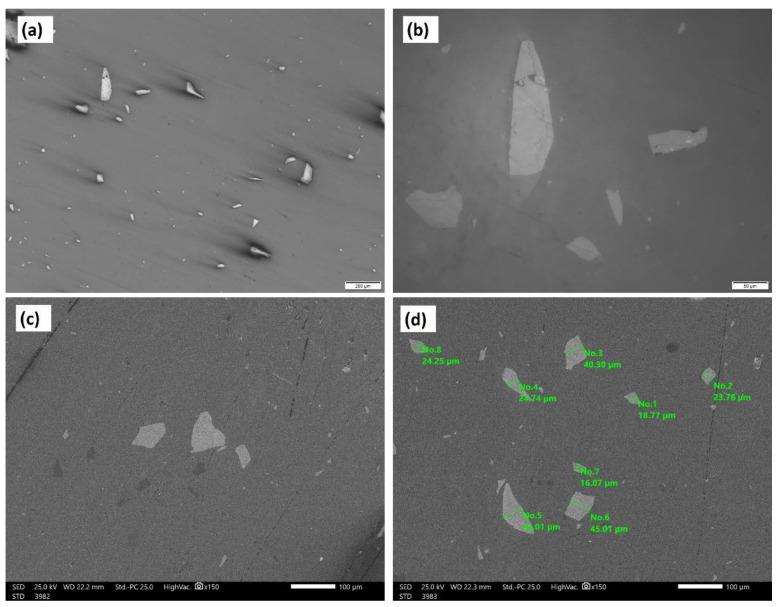
Optical microstructure at different magnifications (**a**,**b**) and SEM microstructure at different positions (**c**,**d**) for the acrylic with 10 wt.% ZrO sample.

**Figure 8:**
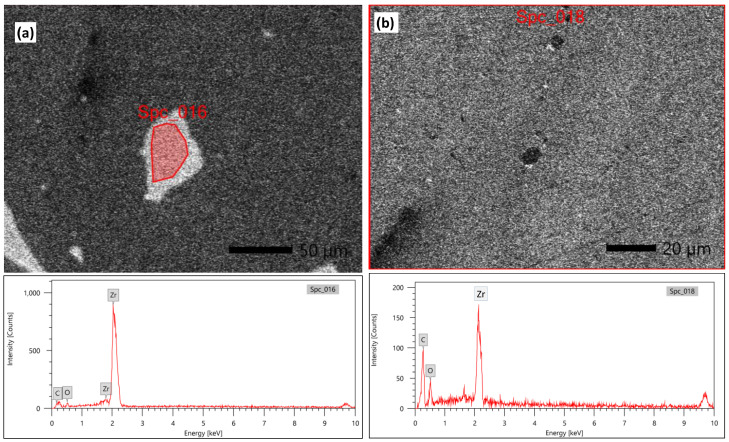
The EDS analysis of the micro-sized particle is presented in (**a**) and the EDS of the nano-composite (**b**).

Figure 5 shows an SEM micrograph of the raw material powder of the ZrO_2_ nanoparticles that were used as reinforcing materials for the acrylic matrix. It can be observed that the powder is of nano-scale, with an almost homogenous particle size that is below 50 nm, except for some agglomerated particles of submicron or micron size. Figure 6 shows the SEM macro and micrographs of the base acrylic material (Figure 6a,b), 5 wt.% ZrO_2_ nanocomposites (Figure 6c,d) and 10 wt.% ZrO_2_ nanocomposites (Figure 6e,f). At the macro level, the samples showed no porosity defects, which indicates the effectiveness of the production procedure at producing sound acrylic nanocomposites at different percentages. On the other hand, the presence of macro-size particles, which increased in line with the increase in the ZrO_2_ weight percentage, can be observed. The sources of these macro-sized particles were the ZrO_2_ particles, as some macro-sized particles were noted within the SEM micrograph of the raw powder. In addition, at the micro-level, no defects were observed either, and nanoparticles can only be observed in the sample of 10 wt.% ZrO_2_ (Figure 6f). Figure 7 shows the optical microstructure at different magnifications (Figure 7a,b) and the SEM microstructure at different positions (Figure 7c,d) for the acrylic with 10 wt.% ZrO sample. The micrographs show a macro-sized particle, with some particle measurements indicated. The EDS analysis of the micro-sized particle is presented in Figure 8a and the EDS of the nano-composite is shown in Figure 8b. The EDS analysis confirmed that the large size particles were mainly ZrO_2_ particles and also confirmed the presence of ZrO_2_ nanoparticles in the acrylic matrix, as indicated in Figure 8b. In addition, the peak carbon can be seen in both EDS charts. This may have been due to the polymeric material and may have resulted from interference from the instrument [38,39].

### 3.2. Micro-Hardness Analysis

**Figure 9:**
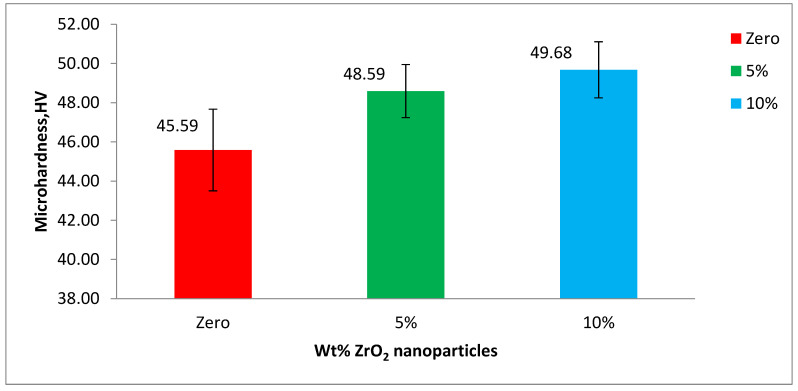
Mean hardness (VHN) of the tested groups of PMMA.

The mean microhardness values and their statistical data were obtained by analysis of variance (one-way ANOVA) at *p*-value < 0.05, for the base acrylic material and the ZrO_2_-reinforced groups. Furthermore, the data are illustrated in Figure 9 as a bar chart of microhardness against the wt.% of the ZrO_2_ nanoparticles. The microhardness of the base acrylic material is 45.59 HV that increased to 48.59 HV after adding 5 wt.% ZrO_2_ nanoparticles. A further increase in microhardness, up to 49.68 HV, was produced by increasing the concentration of the ZrO_2_ nanoparticles to 10 wt.%. 

### 3.3. Wear Behavior 

**Figure 10:**
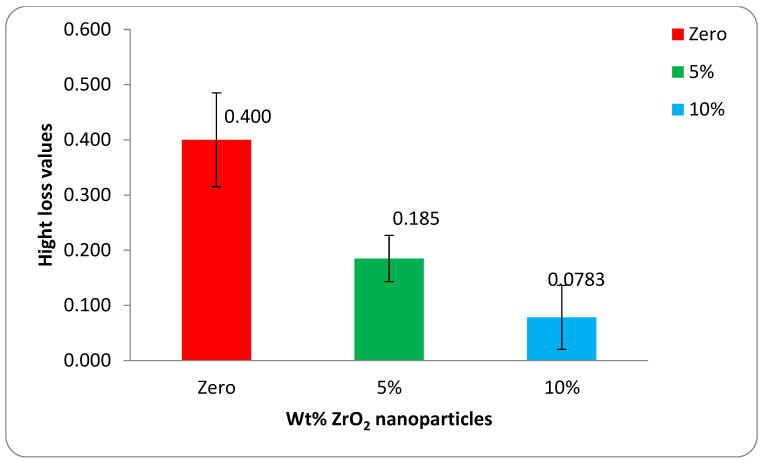
Mean height loss of the tested groups after wear testing.

**Figure 11:**
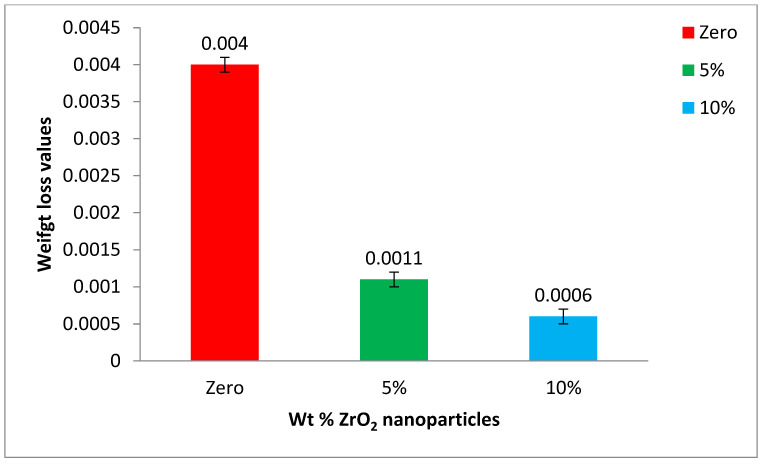
Mean weight loss of the tested groups after wear testing.

The mean height loss values and their statistical data were obtained by analysis of variance (one-way ANOVA) at *p*-value < 0.05 for the base acrylic material and the ZrO_2_-reinforced groups. The data are illustrated in Figure 10 as a bar chart of the mean height loss against the wt.% of the ZrO_2_ nanoparticles. Furthermore, the mean weight loss values and their statistical data were obtained by analysis of variance (one-way ANOVA) at *p*-value < 0.05 for the base acrylic material and the ZrO_2_-reinforced groups. The data are illustrated in Figure 11 as a bar chart of the mean weight loss against the wt.% of ZrO_2_ nanoparticles. The results of the wear tests of the height loss and weight loss both indicate a significant improvement in the wear resistance due to the addition of the ZrO_2_ nanoparticles and that the wear resistance increased by increasing the ZrO_2_ nanoparticle concentration. 

**Figure 12:**
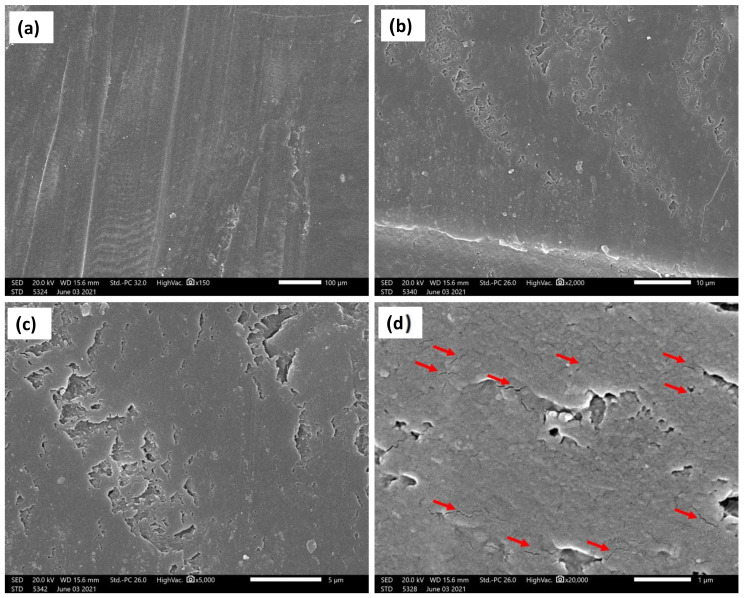
SEM micrographs at different magnifications and positions of the base acrylic material after wear test. Red arrows indicate the microcracks. (**a**) 150×, (**b**)2000×, (**c**) 5000× and (**d**) 20,000×.

**Figure 13:**
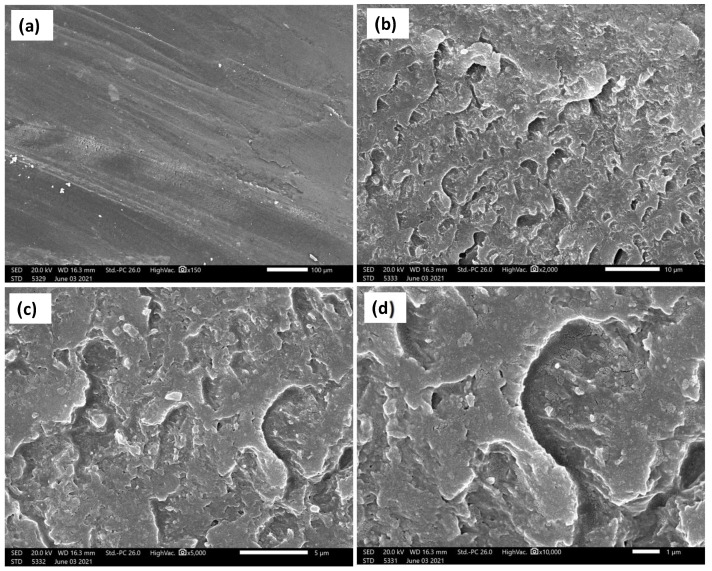
SEM micrographs at different magnifications and positions of the 5 wt.% ZrO_2_ acrylic nanocomposite material after wear test. (**a**) 150×, (**b**)2000×, (**c**) 5000× and (**d**) 10,000×.

**Figure 14:**
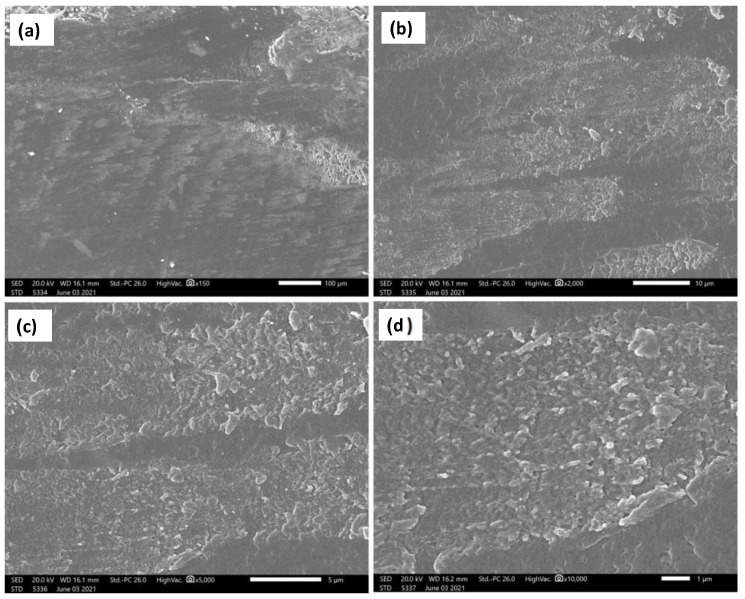
SEM micrographs at different magnifications and positions of the 10wt.% ZrO_2_ acrylic nanocomposite material after wear test. (**a**) 150×, (**b**)2000×, (**c**) 5000× and (**d**)10,000×.

The current investigation showed a significant enhancement of the wear resistance by adding ZrO_2_ nanoparticles. Figure 12, Figure 13 and Figure 14 show the SEM micrographs at different magnifications, (a) 150×, (b) 2000×, (c) 5000×, and (d) 20,000× (Figure 12) and 10,000× (Figure 13 and Figure 14), for the wear surface of the different groups after conducting the wear test. The wear surface of the acrylic base material without ZrO_2_ nanoparticles (Figure 12) showed severe wear features with deep wear tracks that can be observed on the low magnifications micrograph (a) 150×. Furthermore, a high density of microcracks (indicated by red arrows (d) 20,000×) can be observed on the highest magnification micrograph. The incorporation of the ZrO_2_ nanoparticles changed the wear features of the wear surface, as can be observed in Figure 13 for the 5 wt.% ZrO_2_ and in Figure 14 for the 10 wt.% ZrO_2_. Less severe wear features were observed as the smoothness of the surface increased, in line with the increasing ZrO_2_ content. In addition, no microcracks were observed, especially at 10 wt.% ZrO_2_.

## 4. Discussion

Developing new materials with improved properties, especially high wear resistance, is an important focus of research. Thus, this work focuses on the improvement of wear resistance of acrylic denture teeth through the use of nanoparticle filler material. Different weight percentages of ZrO_2_ nanofillers (5% and 10%) were used with the acrylic matrix. The addition of zirconia filler significantly increased the Vicker’s hardness of the tested materials. This may have been due to the characteristics of the ZrO_2_ particles, as well as high interfacial shear strength between the nanofiller and resin matrix, which in turn increased the resistance of the material for penetration [40]. Similar results have been reported in previous studies [9,12]. The increase in microhardness by about 6–8% relative to the base material is similar to that reported in previous studies. Hameed and Abd-Elrahaman [25] investigated the effect of ZrO_2_ nanoparticles on the properties of acrylic denture base and reported an increase of about 5% in microhardness when increasing ZrO_2_ nanoparticles up to 7%.

Wear resistance is an important requirement of prosthetic materials, such as denture teeth, which do not provide a durable functional and esthetic restoration unless they exhibit high wear resistance [41]. Intraoral wear is a complex process related to both the composition of denture teeth and the patient’s oral condition. The ability to replicate and control these factors intraorally is challenging. Therefore, in vitro two-body wear testing is a valid model to measure antagonist wear without involving an intermediate medium that may influence the results [42]. The height and weight loss of the samples were evaluated. In dentistry, height loss is a more clinically relevant parameter, because the vertical distance between the maxilla and mandible is stabilized by the occlusal contact points. However, weight loss provides more accurate measurements, as indicated by lower variation. The wear resistance was significantly improved after the addition of zirconia nanofillers to the polymer matrix at both 5% and 10% concentrations. This result agrees with previous studies, in which concentrations of saline-treated alumina 0.1% [41] and silver nanoparticles 0.2–8% [43] considerably improved the resin wear resistance of PMMA. In terms of the effect of surface roughness on wear resistance, Benli et al. [44] investigated the relationship between the wear rate and surface roughness of occlusal splint materials made of contemporary and high-performance polymers and found that it is directly proportional. Furthermore, Nayyer et al. [45] reported that surface roughness is affected by clinical adjustments such as polishing, because polishing leads to smooth surfaces that undergo less wear and provides the advantage of extended longevity of the restoration [46,47]. It should be mentioned here that in this study, the samples were examined for wear behavior without polishing; thus it was expected that the polishing would significantly improve the wear resistance of the developed nanocomposites.

This improvement can be attributed to the fact that the ZrO_2_ particles exhibited greater hardness than the surrounding resin matrix and, therefore, were not as easily abraded by the antagonist [40]. The SEM of the acrylic base material without ZrO_2_ particles (Figure 12) showed high-density microcracks, which may have been the main mechanism behind the deterioration of the surface and the high reduction in both the height and weight observed in the measurements above. This might indicate that the type of wear was fatigue wear due to the formation of cracks under the subsurface. It has been reported that fatigue wear occurs when microcracks form below the surface of a material and eventually coalesce to free a small fragment of the material. Materials with a lower elastic modulus, such as unfilled resins, are susceptible to fatigue wear [42]. Furthermore, increasing the concentration of ZrO_2_ from 5% to 10% produced more improvement in the wear resistance, which can be attributed to the change in the wear mechanism from fatigue wear to abrasive wear type. This was confirmed by the elimination of microcracks at high ZrO_2_ particle concentrations, as can be observed in Figure 14. The results suggest that wear resistance may be influenced not only by the presence or absence of fillers, but also by the type, amount and size of the filler particles, as well as by how the filler particles are integrated into the matrix. However, the results of this study can provide guidance because the applied in vitro test arrangement cannot fully replicate the intraoral conditions. Although the use of natural enamel as an antagonist is considered the gold standard, disadvantages do exist, including variability in the form, fluoride content, and amount of aprismatic enamel; therefore, standardizing each stylus is not easy [48]. Further in vivo studies are needed to investigate the effect of adding nano ZrO_2_ with different concentrations on wear resistance and other mechanical properties intraorally in acrylic resin denture teeth.

## 5. Conclusions 

This study investigated the effect of using ZrO_2_ nanoparticles as a filler material in acrylic denture teeth, aiming to enhance the wear resistance. Based on the obtained results, the following conclusions can be drawn:

(1) The microstructural investigation of reinforced denture teeth indicates sound material preparation using the applied regime without porosity or macro defects. No segregation was observed for the nanoparticles; only submicron-sized ZrO_2_ particles were found.
(2) The addition of zirconium oxide nanofillers to PMMA at both 5% and 10% increased the microhardness, with values up to 49.7 HV found after the addition of 10 wt.% ZrO_2_.
(3) The wear mechanism in the acrylic base material without nanoparticle addition was found to be fatigue wear; a high density of microcracks was found.
(4) The addition of 5 wt.% ZrO_2_ significantly reduced weight loss and height loss relative to the base material, which indicates improved wear resistance.
(5) Increasing the nanoparticles to 10 wt.% ZrO_2_ further improved the wear resistance, with no microcracks found.

## Data Availability

This will be made available upon request through the corresponding author.
